# Energy Conservation Using Dynamic Voltage Frequency Scaling for Computational Cloud

**DOI:** 10.1155/2016/9328070

**Published:** 2016-04-28

**Authors:** A. Paulin Florence, V. Shanthi, C. B. Sunil Simon

**Affiliations:** ^1^Sathyabama University, Chennai 600 119, India; ^2^St. Joseph's Institute of Technology, Chennai 600 119, India; ^3^St. Joseph's College of Engineering, Chennai 600 119, India; ^4^Zoho Corporation, Chennai, India

## Abstract

Cloud computing is a new technology which supports resource sharing on a “Pay as you go” basis around the world. It provides various services such as SaaS, IaaS, and PaaS. Computation is a part of IaaS and the entire computational requests are to be served efficiently with optimal power utilization in the cloud. Recently, various algorithms are developed to reduce power consumption and even Dynamic Voltage and Frequency Scaling (DVFS) scheme is also used in this perspective. In this paper we have devised methodology which analyzes the behavior of the given cloud request and identifies the associated type of algorithm. Once the type of algorithm is identified, using their asymptotic notations, its time complexity is calculated. Using best fit strategy the appropriate host is identified and the incoming job is allocated to the victimized host. Using the measured time complexity the required clock frequency of the host is measured. According to that CPU frequency is scaled up or down using DVFS scheme, enabling energy to be saved up to 55% of total Watts consumption.

## 1. Introduction

Today the world's mantra is big data. Every domain requires deft handling of voluminous data for their survival with example being social networking sites like Facebook, stock exchanges, online shopping portals, travel portals, hospital management, government sectors, and so forth. In order to process data they depend on computational resources which need a huge amount of capital expense. Hence all organizations are moving towards cloud.

With every passing day, world is waking up to the importance of staying connected seamlessly and this is becoming possible only with cloud computing enabled devices making its way into the work and personal world enabling us to continue the work from where it is left. Cloud computing works on principle of two slogans “Pay as you go” and “Anything as a Service (AaaS)” [1]. For example, as citizen of a country, you can consume power by connecting yourself with a service provider and have to pay only for as much power as you consume. However any resource like infrastructure, software, and network platform can be availed as a service from the cloud.

Cloud caters IaaS, SaaS, PaaS, and so forth and facilitates dynamic resource provisioning [1]. It meets fluctuating demand by varying the resources and service requirement needs of customers. In order to provide reliable service it is essential to structure Service Level Agreement (SLA) [2] and also Quality of service (QoS) [3]. SLA is a legal binding contract which encompasses guaranteed QoS that an execution environment (provider) agrees to provide to its hosted application. It should be noted that every service provider has to fulfil the obligation of the client based on their SLA and provide complete service. Some of the characteristics of customer requirements can be summed up by QoS [3] among which the most important are availability of services, security, and reliability.

It can also be noted that QoS is fundamental for cloud users [2]. Cloud service providers have to find the right tradeoffs between QoS levels and operational costs together with which they are able to deliver the advertised quality characteristics [3].

Availing services from cloud reduce capital expenditure of an organization [4]. There are varying numbers of cloud providers offering dynamic resource provisioning. Cloud data centers consume enormous amount of power and in turn generate large amount of heat which poses massive impact on environmental conditions [4] and inflates power bill.

Many approaches are devised to address this major concern; however it has its own repercussion. Trying to operate a processor at lower power state degrades the overall performance and this impacts the output.

To ensure optimal performance of servers and at the same time ensure quality of output, we have devised a new methodology which studies the pattern of the incoming request, categorizes the algorithm adopted, identifies its time complexity using asymptotic notations, and applies a new approach which saves energy consumption.

## 2. Related Work

Lin et al. [5] proposed a novel algorithm for the mobile cloud computing (MCC) task scheduling problem to minimize the total energy consumption of an application in a mobile device. It generates a task schedule and then migrates the task either to the local core or to the cloud to reduce energy consumption. It identifies tasks that need to be offloaded onto the cloud and then forwards the remaining tasks to the local core and further tries to reduce energy consumption based on execution time requirements of each task by applying DVFS. It focuses mainly on battery-powered devices and tries to reduce scheduling delay involved in offloading the tasks onto the cloud. A linear-time rescheduling algorithm is proposed for task migration.

Two-layer control architecture called EvGPU (energy-efficient SLA guarantees virtualized GPU) is devised by Guan et al. [6] for cloud gaming. At the first layer of the control architecture the author has implemented Proportional-Integral (PI) controller which guarantees SLA and measures the number of Frames Per Second (FPS) for each online game based on the set threshold level. Then at the second level to reduce power consumption GPU frequency is adjusted through DVFS based on the current FPS. Hence EvGPU could dynamically assign virtual GPU resources for cloud gaming. This approach helps in reducing power consumption of GPU through DVFS with a given GPU utilization or a given workload.

Jeyarani et al. [7] propose a power aware meta scheduler which predicts the incoming VM request pattern based on recent history of arrivals and allocates resources. To conserve power, adaptive provisioning policy along with power aware allocation policy and chip aware VM scheduler is adapted. Here PEs are transitioned between shallow power saving state to deep sleep state. This system fails to identify exact request pattern, because it follows arrival history of VM request in order to allocate resources.

 Quan et al. [8] devised an algorithm to optimize resource allocation and workload consolidation and finally frequency of the core gets adjusted. This is achieved by moving heavy load applications to new servers with great number of cores with an assumption that new generation computer components have higher performance and consume less energy than the old generation and by also moving light load applications to old servers with less number of core servers and switch off the idle old servers.

## 3. Methodology for Energy Conservation Using Dynamic Voltage and Frequency Scaling for Computational Cloud

The reason why organizations are moving to cloud rather than establishing their own infrastructure is to save money. They can share their geographically distributed resources. But processing big data has its own disadvantages. It involves usage of lot of power and every unit consumed will have a direct impact on the balance sheet of a company and secondly when the use of power increases, heat generation increases, which is hazardous to the environment. Besides it also reduces the efficiency of electrical components, which is inversely proportional to increasing temperature due to the deteriorating state of those components. For the aforementioned reasons, we try to find a way to reduce the energy consumed at data centers [4].

### 3.1. Architecture Diagram

Common user performs an action (e.g., search), which is grabbed as a request by any random broker or whoever providing that service. As it could be any unknown broker, the broker can use any kind of unknown algorithm at one end to have that request executed from within cloud systems. Hence he fits that request inside his own framework, which is redirected to the Cloud Operational Control Center (COCC). Thus now it is the job of the COCC to analyze the resource requirements of that unknown algorithm by means of identifying the type of that algorithm and execute it as per resource availability and by manipulating better energy utilization.

**Figure 1:**
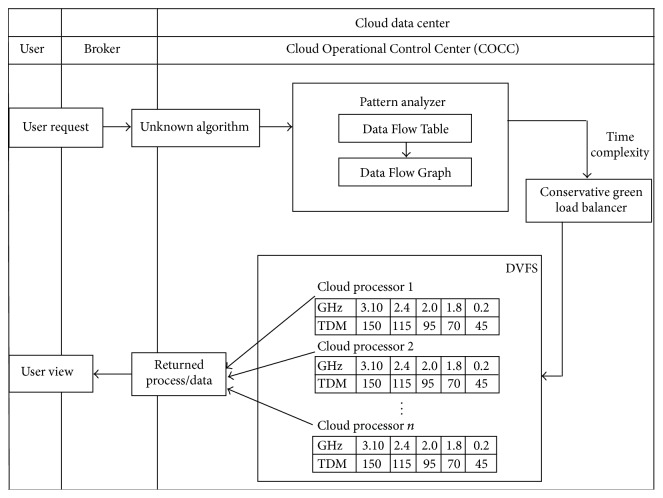
Architecture diagram.

COCC is comprised of three components such as pattern analyzer, green conservative load balancer, and DVFS. The pattern analyzer analyzes the incoming unknown algorithm and constructs Data Flow Table (DFT) which in turn extracts the algorithm pattern which is depicted as Data Flow Graph (DFG). The DFG helps to derive the type of algorithm and computes the time complexity. Green load balancer locates the most promising node to execute the user request based on the computed time complexity. Then the frequency of the victimized node is adjusted as per the requirement.  Figure 1 shows the overall view of system architecture.

## 4. Discussion

In cloud, requests are to be served efficiently which in turn enables optimal utilization of power. Since power plays a very vital role in the way entire cloud computing architecture operates, it is quiet critical that optimization or in other terms minimal utilization of power is important.

Various strategies are developed to reduce power consumption and one of them is the DVFS scheme which can be used to minimize the overall power consumption, thus enabling cloud computing to work effectively [5]. DVFS scheme facilitates frequency adjustment of processor according to the need, where the processor has to operate at its full speed and vice versa.

DVFS is a power conservation technique [9], whereby CPU frequency can be dynamically scaled according to its load [10]. DVFS can be activated in 4 different modes [11]:

- high frequency,
- low frequency,
- available frequency for the CPU,
- on demand dynamically choosing the frequency level.

Jobs arriving to the cloud may be CPU bound, I/O, or Memory bound. If the job request is CPU bound, the frequency of the processor needs to be adjusted to its required level. If it is I/O bound it needs to be run at its least frequency rate. Hence it becomes important to classify the request as CPU bound or I/O or Memory bound.

The entire incoming request follows one or the other algorithm. Basically every algorithm falls under certain class. From increasing order of growth they are classified as constant time algorithm, logarithmic algorithm, linear-time algorithm, polynomial time algorithm, and exponential time algorithm.

Formally, the complexity of any algorithm can be represented using asymptotic notation. It is classified into *θ* (theta), *O* (Big *O*), and *Ω* (omega) where *O*(*g*(*n*)) establishes an upper bound on the function, *θ*(*g*(*n*)) defines two functions that bound the function *g*(*n*) from both upper and lower limits, and *Ω*(*g*(*n*)) defines a lower bound of the function [12, 13].

However in this paper we have constrained on using only Big *O*'s worst-case time complexity [13]. Hence the processor can be used and optimized according to its requirement. We have devised a methodology which analyzes the behavior of the algorithm coming as a request. Its behavioral pattern is recorded in a DFT and using that DFT the corresponding DFG is derived, and then the fitness of the incoming request is tested with mathematical or procedural functions using the data retrieved from the DFG and once it satisfies a certain algorithm its corresponding time complexity is derived using asymptotic notation. Then total micro instruction sets clock cycles specific to that algorithm which is evaluated and then multiplied with its asymptotic range. A constant variance error variable is added to it for more precision. Based on these requirements we apply DVFS and adjust the host's voltage and CPU frequency according to the requirement.

With the assumption that every request that comes to the cloud follows a particular algorithm, the following algorithms which are generally in use under different domains such as financial markets, networking, artificial intelligence, gaming, and medical are considered:

(i) linear algorithm,
(ii) geometric algorithm,
(iii) backtracking algorithm,
(iv) *K*-means clustering algorithm,
(v) Warshall and Floyd's algorithm,
(vi) binary search algorithm,
(vii) merge algorithm.

### 4.1. Phases of the Proposed Work

This paper focuses on energy conservation in a way of classifying jobs according to their behavioral pattern. It involves three phases. They are

- pattern analyzer,
- conservative green load balancer,
- DVFS implementation.

#### 4.1.1. Pattern Analyzer

In our work we focus on efficient utilization of cloud resources along with energy conservation. Pattern analyzer reads the incoming cloud request (cloudlet) which is framed as an algorithm by the broker and constructs Data Flow Table (DFT), from which it derives its DFG. The flow of the iterators from its pattern of DFG is analyzed and algorithm followed by the request is identified. Its data size based time complexity and micro instruction clock cycle (MICC) count time complexity are calculated following which Million Instructions Per Second (MIPS) is computed. Algorithms considered for analysis are discussed below.

*Linear Algorithm*. One of the widely used search algorithms is linear search algorithm. It is a kind of brute force search. Its time complexity is defined as linear denoted by *O*(*N*). Upon receiving the incoming job request, our classification algorithm studies the flow of the incoming pattern, if it is found to be single loop from 1 to *N*, it identifies that the algorithm is linear [14, 15]. The pattern is shown in Table 1(a).

*Geometric Algorithm*. Our classification algorithm after analyzing the incoming request finds the value between any two successive iterations, and if the multiplicative progression ratio between them is the same, then it identifies that the particular algorithm is geometric algorithm [14, 15]. Its pattern is shown in Table 1(b).

*Backtracking Algorithm*. By analyzing the incoming request, our algorithm decides it is a backtracking algorithm when it finds that the iterator builds candidates to solution incrementally; it prunes and backtracks as soon as it decides that the candidate cannot be a valid solution [14, 15] which is shown in Table 1(c).

*K-Means Clustering Algorithm*. *K*-means is one of the famous clustering algorithms in data mining. *K*-means clustering aims to partition *n* number of observations into *k* number of clusters in which each observation belongs to the cluster with the nearest mean. Each subsequent element is put into *k* clusters and individual means are updated simultaneously by finding which element is near to which mean and eventually dropped into that cluster [14, 15]. The pattern thus derived is shown in Table 1(d).

*Warshall and Floyd's Algorithm*. Warshall's is a graph analysis algorithm that is used to identify existence of a path, while Floyd's is a graph analysis algorithm for finding all pairs shortest path using a weighted graph with positive or negative edge weights. A single execution of the algorithm returns the summed lengths or summed weights of the shortest paths between* all* pairs of vertices [14, 15]. Its pattern is depicted in Table 1(e).

*Binary Search Algorithm*. Binary search is also known as half-interval search algorithm that finds the position of a searched value within an array that has to be arranged in ascending or descending order. In each step, the algorithm compares the search value with the value of the middle element of the array and accordingly adjusts its bound limits for subsequent searches until the element is found and then its position is returned [14, 15]. The pattern attained from DFT is shown in Table 1(f).

*Merge Algorithm*. Merge algorithm is a divide and conquer type algorithm that runs sequentially over unsorted lists and accordingly adjusts its bound limits for subsequent merges typically producing more sorted lists as output by means of breaking down and joining back [14, 15]. The pertaining pattern is shown in Table 1(g).

The patterns of various algorithms derived as a result of analysis are shown in Table 1.

*(1) Computation of Time Complexity*. Once pattern analyzer ascertains the type of algorithm shadowed by the incoming cloudlet, time complexity is evaluated by using two techniques named “data size based time complexity” and “micro instruction clock cycle count time complexity.” Data size based time complexity of an algorithm's performance time may vary with different input size. In our case, we have used worst-case time complexity of Big *O* notation [13], denoted as *O*(*T*(*n*)), which is defined as the maximum amount of time taken on any input of size *n*.

The data size based time complexities by the nature of the function *O*(*T*(*n*)) considered for experimental purpose are shown in Table 2.

After data size based time complexity is evaluated its micro instruction clock cycle is derived. Each algorithm is composed of a particular set of instructions. Each instruction execution is a four-process cycle called machine cycle which involves the following steps:

1. instruction fetch cycle (IF),
2. instruction decode cycle (ID),
3. execution cycle (EX),
4. write cycle (WB).

Each instruction cycle is comprised of micro instructions referred to as micro program. Micro instruction clock cycle count time complexity means clock pulses needed for individual instruction cycle that is accumulated for the entire code and accordingly total clock cycle consumption is derived. Hence the MIPS estimation of the algorithm is achieved by multiplying the data size based time complexity of the identified algorithm with its corresponding MICC which is added with a constant for more precision subsequently.

**Algorithm 1 (pattern analyzer).**

 

*Input*. Incoming algorithm (cloud request) called cloudlet.

*Output*. MIPS of incoming cloudlet.

*Step  1*. Read cloudlet.

*Step  2*. Construct Data Flow Table (DFT).

*Step  3*. Read DFT and construct Data Flow Graph (DFG).

*Step  4*. Evaluate the pattern followed in DFG and find out the algorithm and its data size based time complexity.

*Step  5*. Assume micro instruction clock cycle [MICC] for the derived algorithm.

MIPS = (time complexity [algorithm] *∗* MICC [Algorithm]) + *c*, where *c* is a constant for error deviation.

**Figure 2:**
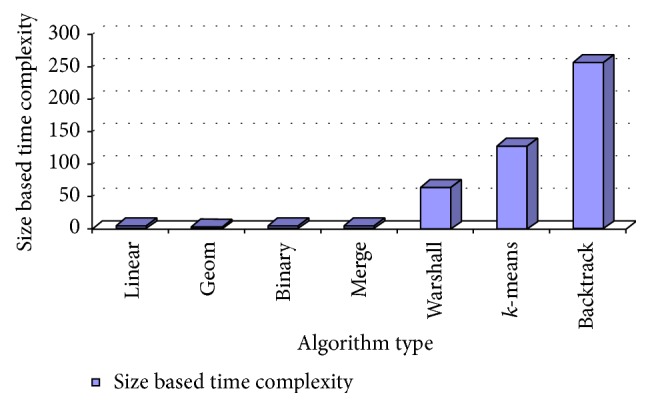
Size based time complexity of various algorithms.

Considering the following approximation values, size based time complexities of some of the algorithms are analyzed and it is shown in Figure 2:

 data search size (*n*) = 4 (in millions),
 Warshall's number of network edges (*v*) = 4 (in millions),
 geometric ratio = 2,
 log of base 2 only,
 
*k*.means dimension = 1,
 
*k*.means *k* clusters = 2.

#### 4.1.2. Conservative Green Load Balancer

In the cloud environment the machines are registered with Cloud Information System (CIS) regarding their characteristics such as their individual id, ram size, and machine name For simulation purpose we have considered 8 virtual machines with MIPS capacity of 3100 MHz and a 1 GB RAM for each of them is configured and registered with the CIS under one common data center. The broker accesses the CIS for the execution of their with respect to cloudlets using their registered information. The cost effective efficiency of a data center depends on the optimal balancing of incoming cloudlets from the queue for execution over available machines. In order to utilize the resources optimally without having to lose efficiency, green conservative load balancing strategy is implemented on the basis of their executional length in terms of millions of instruction execution strength per second (exe_length). When a request comes in for execution over cloud, the request is diverted for execution as per its requirements by means of best fit strategy available from among the overall availability of each virtual machine's idle clock pulse (mach[*m*].capacity).

This green load balancer which adapts “best fit strategy” looks for an appropriate VM and adjusts its frequency by applying DVFS. Thus the VM is allowed to operate at the required clock frequency rather than in its defined speed.

**Algorithm 2 (green load balancer).**

 

*Input*. Cloudlet MIPS.

*Output*. Victimized VM, required frequency scale.

*Step  1*. Let *q* = 0.

*Step  2*. Let *m* = 0.

*Step  3*. If cloudlet[*q*].exe_length less than mach[*m*].capacity then go to Step  4 else go to Step  8.

*Step  4*. Assign Cloudlet[*q*] to mach[*m*].

*Step  5*. mach[*m*].capacity = mach[*m*].capacity-cloudlet[*q*].exe_length.

*Step  6*. Call DVFS.

*Step  7*. Go to Step  10.

*Step  8*. *m* + +.

*Step  9*. If *m* < 8 then go to Step  3.

*Step  10*. *q* + +.

*Step  11*. If *q* < *n* then go to Step  3.

*n* is number of cloudlets, *m* is number of virtual machines, and *q* is number of cloudlets.

#### 4.1.3. DVFS Implementation

Thermal Design Power (TDP) in Watts is the energy produced in terms of heat [16]. The energy in terms of heat is derived by multiplying Volts with ampere; that is, the amount of work (Watts) that can be done depends on both the amount (Amps) and the pressure of the electricity (Volts) where Watts = Volts × Amps. Hence Volts is directly proportional to Watts; for instance, as Volt increases Watts increases automatically. Thus by using DVFS scheme, voltage can be adjusted. Undervolting can be done in order to conserve power and efficient use of cooling devices. Overvolting can also be done in order to increase computing performance.

Now DVFS methodology is applied individually to the victimized processor, therefore reducing the overall Watts needed as generated by each request, and thus dynamically adjusts by scaling the MIPS frequency of the processor and optimizing the use of electricity by fluctuating it as per required and also reduces the voltage [9].

**Algorithm 3 (DVFS).**

 

*Input*. Victimized VM, required frequency scale.

*Output*. Optimized energy consumption in Watts

*Step  1*. DVFS = 0.

*Step  2*. If (3.1-mach[*m*].capacity) < range [dvfs] then go to Step  3 else go to Step  5.

*Step  3*. Scale the vm to range [dvfs].

*Step  4*. Go to Step  7.

*Step  5*. dvfs++.

*Step  6*. If dvfs < 5 then go to Step  2.

*Step  7*. Return.

## 5. Experimental Section

For simulation, we have considered Facebook's new data center in Lulea, Sweden. It has nearly 60000 servers and each server uses two Intel Xeon Processors 3.10 GHz which needs 150 Watts of energy. Therefore per server 300  Watts of power is essential, which is nearly 18000000 W or 18 MW power. It is necessary just for processors out of 120 MW for the entire 3000000 sqfeet plant [17]. This is nearly 15% of the entire consumption. We have implemented our DVFS strategy based on 5-level scaling, that is, at 3.1 GHz, 2.4 GHz, 2 GHz, 1.8 GHz, and 0.2 GHz of 2600 series Intel Xeon Processors.

Table 3 shows 5 level scaling options with respect to their Watts energy requirement configured by Intel [18].

**Figure 3:**
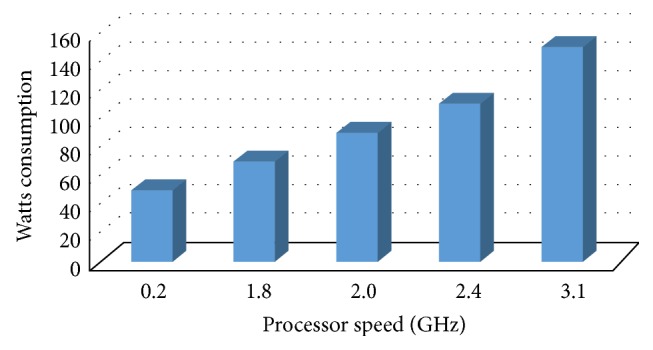
Standard Watts consumption of different processors.

Figure 3 shows standard Watts consumption of different processors at different speeds.

### 5.1. Consideration for Experimental Purpose

#### 5.1.1. Pattern Analyzer

 Algorithm identified is linear search algorithm,
 Time complexity for linear search algorithm is *O*(*n*)
 Assume that MICC as per linear algorithm for our purpose consumes 50 clock cycles:
  (i) therefore *O*(100000) = 100000,
  (ii) time taken to execute one microprogram in MIPS = 50,
  (iii) MIPS = size based time *∗* MICC + *c*,
   = 100000 *∗* 50 + 0 (as for now *c* is constant 0 error deviation)
   = 5000000 (5 million clock cycles).

### 5.2. Conservative Green Load Balancer

 Processor type is 3.1 GHz processor.
 Data set size (*n*) is 100000 million.
  (i) 3.1 GHz machine = 3.1 billion clock cycles per second.
  (ii) Therefore (3.1 billion − 5 million clock cycles) = 2.95 billion clock cycles unused.

### 5.3. DVFS

Scale the machine to .2 GHz, which is sufficient to execute 5000000 (5 million clock cycles).

The 2.95 remaining clock cycles can be used for incoming cloudlets.

## 6. Results

**Figure 4:**
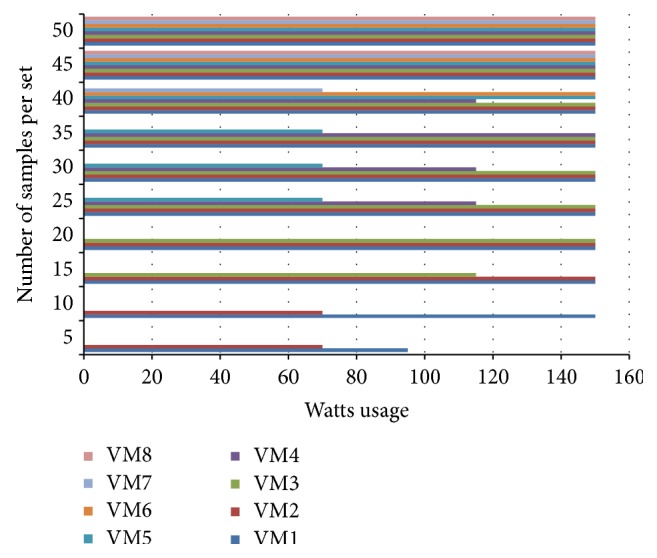
Load on 8 virtual machines for randomly generated requests.

Load on eight virtual machines recorded over 50 times randomly generated sets of 5, 10, 20, 25, 30, 35, 40, 45, and 50 and requests are shown in Figure 4, which eventually depict how load is balanced among the virtual machines.

**Figure 5:**
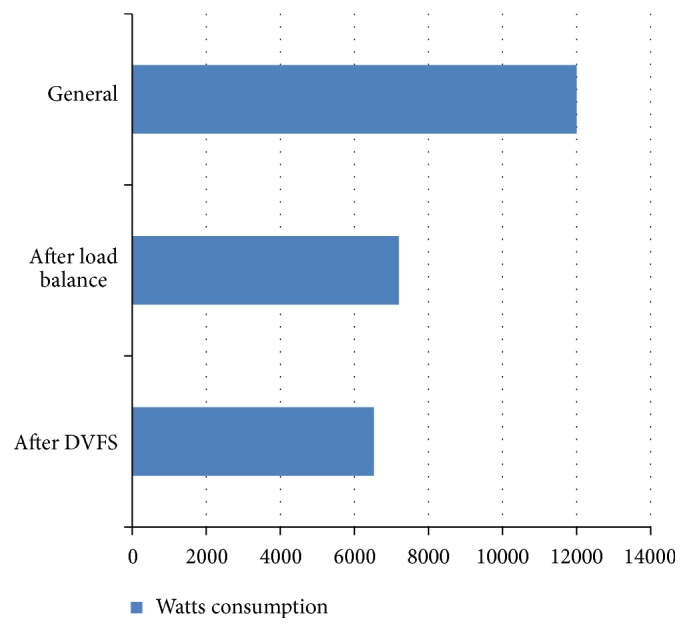
Watts consumption.

Figure 5 displays the energy requirement statistics from a simulation model of randomly generated test case of 5, 10, 20, 25, 30, 35, 40, 45, and 50 into our proposed research model. The horizontal axis shows Watts consumption range and the vertical axis calibrates the three-stage test such as using conservative model, load balance before applying DVFS, and load balance after applying DVFS.

It is obvious from the graph that the energy consumption has drastically reduced to a higher extent from 12000  Watts to 6500  Watts after the full implementation of our research model. Thus our extensive simulation shows the effectiveness of our research proposal model that saves and reduces the energy consumption to a phenomenal rate.

## 7. Conclusion

This work analyses the incoming cloud task request, studies the flow pattern of the task, and identifies the algorithm, hence computing the time complexity based on asymptotic notation and MICC. So by considering the time complexity this new approach victimizes a VM whose frequency best fits the incoming task and subsequently scales the frequency of the VM to the required level, thus allowing the VM to operate at its desired frequency. Simulation results exhibit efficiency of the proposed approach. This technique can be used for creating custom built cooling systems control, which can dynamically work together for more accurate power saving methodologies. Likewise processors with finer scaling capacities can be built on the basis of this technique in order to attain more flexibility in frequency scaling.

## Figures and Tables

**Table 1:** Derived Data Flow Graphs with respect to their algorithm patterns.

Type	Data Flow Graph (DFG)	Algorithm
Linear	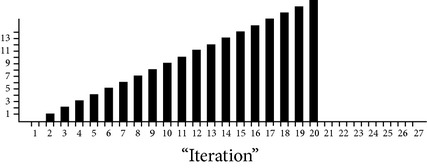 (a) Linear	**If ** *alg* is unknown then *assume alg* is linear algorithm **for **shift_*h* ← 1 to *n* − 2 then **if **(*table*[shift_*v*][shift_*h* + 2])−(*table*[shift_*v*][shift_*h* + 1]) not equals (*table*[shift_*v*][shift_*h* + 1]) − (*table*[shift_*v*][shift_*h*])) then *alg* is unknown **break** end if end for end if

Geometric	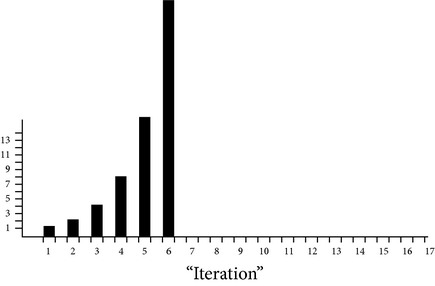 (b) Geometric	***If*** * alg is unknown then* *Assume alg is a geometric algorithm* ***For*** * shift_h* *←* *1 to n − 2 then* ***If*** * (table[shift_v][shift_h + 2])/(table[shift_v][shift_h + 1])* *not equals (table[shift_v][shift_h + 1])/(table[shift_v][shift_h]) then* *alg is unknown* ***break*** **end if** **end for** **end if**

Backtracking	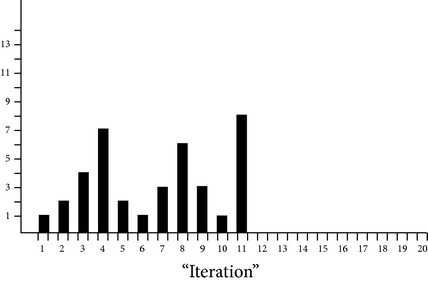 (c) Backtracking **If ** *alg* is unknown then *Assume alg* is a backtraking algorithm **For **shift_*h* ← 1 to *n* then **If ** *table*[shift_*v*][shift_*h*] is greater than *n* then *alg* equals unknown shift_*h* equals *n*	**Break** **end if** **If ** *table*[shift_*v*][shift_*h*] is equals to 1 then *alg* equals unknown shift_*h* equals *n* **break** **end if** **if ** *table*[shift_*v*][shift_*h*] equals *table*[shift_*v*][shift_*h* − 1] *alg* equals unknown shift_*h* equals *n* **break** **end if** **for ** *k* ← shift_*h* − 1 *k* greater than 1 and *k*− − then **if ** *table*[shift_*v*][*k*] equals *table*[shift_*v*][shift_*h*] then **for ** *p* ← *k* + 1 to shift_*h* then back[*table*[shift_*v*][*p*]] equals 1; end for *k* equals −1; end if end for end if end for

*K*-means clustering	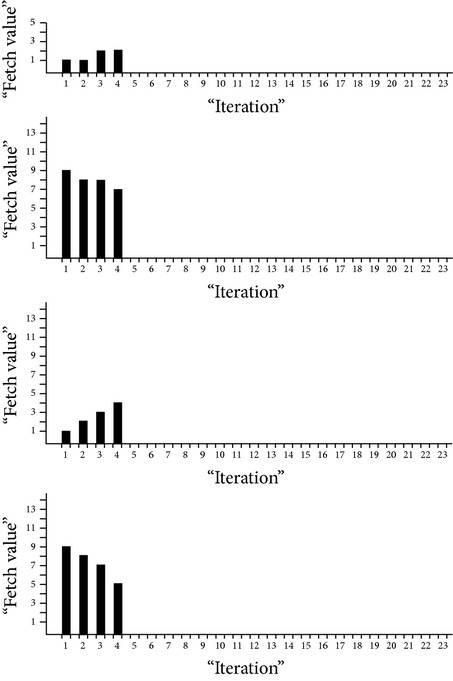 (d) *K*-means clustering	*k*_mean_tot equals 0; **if ** *alg* equals unknown then * assume alg* is a *k* means algorithm **for** *k* ← 0 to 2 **for** *p* ← 1 to *n* − 1 *k*_mean_tot equals 0 **for **shift_*h* ← 1 to less than or equals *p* *k*_mean_tot equals *table*[*k*][shift_*h*] + *k*_mean_tot; end for **if ** *k*_mean_tot/*p* not equals *table*[*k* + 2][*p*] then *alg* is unknown *p* equals *n* *k* equals 2 **break** end if end for end for end if

Warshall and Floyd's	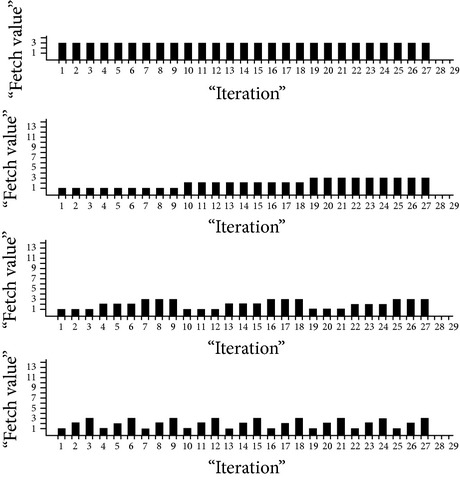 (e) Warshall and Floyd's	If alg is unknown then Assume alg is warshal/floyd For shift_*v* ← 0 to end of data flow table shift_*h* equals 1; While table[shift_*v*][shift_*h*] not equals end of line If table[shift_*v*][shift_*h*] greater than no. of edges^∧^3 then alg equals unknown break end if shift_*h*++ wend If shift_*h* − 1 not equals no. of edges^∧^3 then alg equals unknown Break End if End for End if

Binary	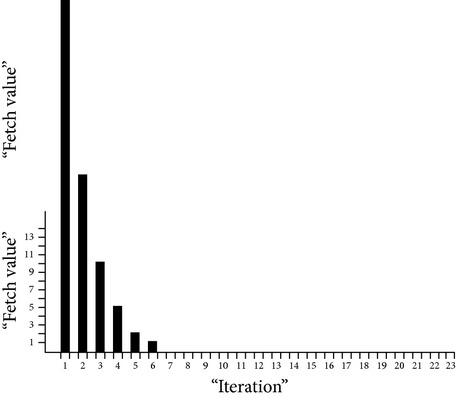 (f) Binary	**If ** *alg* is unknown then *Assume alg* is binary **For **shift_*h* ← 1 to less than *n* − 1 then **if** (*table*[shift_*v*][shift_*h* + 1]) not equals (*table*[shift_*v*][shift_*h*])/2) *alg* is unknown **break** end if end for end if

Merge	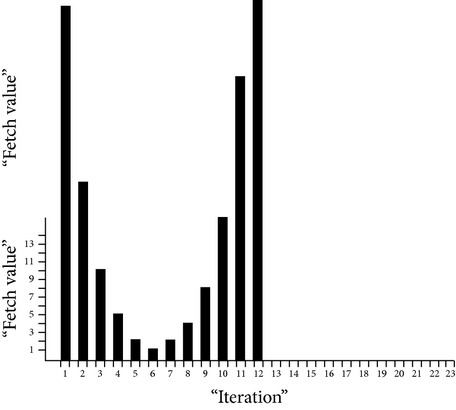 (g) Merge	**If ** *alg* is unknown *Assume alg* is a merge algorithm **for **shift_*h* ← 1 to less than (*n* − 1)/2 **If** (*table*[shift_*v*][shift_*h* + 1]) not equals (*table*[shift_*v*][shift_*h*])/2 *alg* is unknown **break** end if end for **for **shift_*h* ← (*n* − 1)/2 to less than *n* − 1 **if** (*table*[shift_*v*][shift_*h* + 1]) not equals (*table*[shift_*v*][shift_*h*])*∗*2) *alg* is unknown **break** end if end for end if

**Table 2:** Algorithm and time complexity.

S. number	Algorithm type	Worst-case function *T*(*n*)	Variables and their definitions	Mathematical functions
1	Linear algorithm	*O*(*n*)	Number of entities to be linear-searched	((*n* + 2)(*n* − 1))/2*n* For all *n* when *k* = 0 or (*n* + 1)/(*k* + 1) when *k* is 1 to *n*

2	Binary search algorithm	*O*(*n*)	*n* is the number of entities to be binary-searched	2^*x*^ = *n* , where *x* = log_2_⁡*n*

3	Geometric algorithm	*O*(*n*/gr)	gr is a constant geometric multiplier and *n* is the number of entities	*a*_*n*_ = *r* · *a*_*n*−1_ for *n* ≥ 1

4	Merge algorithm	*O*(*n*log⁡(*n*))	Number of entities to be merged	$T(n)=2T\left(\frac{n}{2}\right)+n$

5	Warshall and Floyd's algorithm	*O*(|*V*|^∧^3)	*V* is the number of vertices within a network	Shortestpath(*i*, *j*, *k* + 1) = min⁡(shortestpath(*I*, *j*, *k*), shortestpath(*i*, *k* + 1, *k*) + shortestpath(*k* + 1, *j*, *k*))

6	Backtracking algorithm	*O*(*n*!)	*n* is the number of entities to be backtracked	$\arg\min\underset{s}{\sum }\overset{k}{\underset{i=1,x\in s_{i}}{\sum }}{\left\|x-\mu \right\|}^{2}$

7	*k*-means clustering algorithm	*O*(*n* ^∧^(*dk* + 1)) | *O*(*g*(*n*))	*k* and *d* (the dimension) are fixed, the problem can be exactly solved, where *n* is the number of entities to be clustered	$m_{i}^{\left(t+1\right)}=\frac{1}{\left|S_{i}^{\left(t\right)}\right|}\underset{x_{j}\in S_{i}^{\left(t\right)}}{\sum }x_{j}$

**Table 3:** Watts consumption of 5-level scaling.

Vendor	MIPS	Cache and series	Cores	TDP
Intel® Xeon® Processor E5-2687W	3.1	20 M, GT/s	8	150
Intel® Xeon® Processor E5-2665	2.4	20 M, GT/s	8	115
Intel® Xeon® Processor E5-2650	2.0	20 M, GT/s	8	95
Intel® Xeon® Processor E5-2648L	1.8	20 M, GT/s	8	70
Intel® Pentium pro	0.2	1.12 M	4	45
